# Global metabolic changes induced by plant-derived pyrrolizidine alkaloids following a human poisoning outbreak and in a mouse model†
†Electronic supplementary information (ESI) available. See DOI: 10.1039/c6tx00221h


**DOI:** 10.1039/c6tx00221h

**Published:** 2016-08-12

**Authors:** Oliver Robinson, Mireille B. Toledano, Caroline Sands, Olaf Beckonert, Elizabeth J. Want, Rob Goldin, Michael L. Hauser, Alan Fenwick, Mark R. Thursz, Muireann Coen

**Affiliations:** a MRC-PHE Centre for Environment and Health , School of Public Health , Imperial College London , UK; b ISGlobal , Centre for Research in Environmental Epidemiology (CREAL) , Spain; c Hospital del Mar Medical Research Institute (IMIM) , Barcelona , Spain; d CIBER Epidemiología y Salud Pública (CIBERESP) , Spain; e Computational and Systems Medicine , Department of Surgery & Cancer , Faculty of Medicine , Imperial College London , UK . Email: m.coen@imperial.ac.uk; f Department of Medicine , Imperial College London , UK; g One Health Foundation , Switzerland; h Schistosomiasis Control Initiative , School of Public Health, Imperial College , London , UK

## Abstract

Several hundred cases of Hirmi Valley Liver Disease (HVLD), an often fatal liver injury, occurred from 2001 to 2011 in a cluster of rural villages in Tigray, Ethiopia. HVLD is principally caused by contamination of the food supply with plant derived pyrrolizidine alkaloids (PAs), with high exposure to the pesticide DDT among villagers increasing their susceptibility. In an untargeted global approach we aimed to identify metabolic changes induced by PA exposure through ^1^H NMR spectroscopic based metabolic profiling. We analysed spectra acquired from urine collected from HVLD cases and controls and a murine model of PA exposure and PA/DDT co-exposure, using multivariate partial least squares discriminant analysis. In the human models we identified changes in urinary concentrations of tyrosine, pyruvate, bile acids, *N*-acetylglycoproteins, *N*-methylnicotinamide and formate, hippurate, *p*-cresol sulphate, *p*-hydroxybenzoate and 3-(3-hydroxyphenyl) propionic acid. Tyrosine and *p*-cresol sulphate were associated with both exposure and disease. Similar changes to tyrosine, one-carbon intermediates and microbial associated metabolites were observed in the mouse model, with tyrosine correlated with the extent of liver damage. These results provide mechanistic insight and implicate the gut microflora in the human response to challenge with toxins. Pathways identified here may be useful in translational research and as “exposome” signals.

## Abstract 2

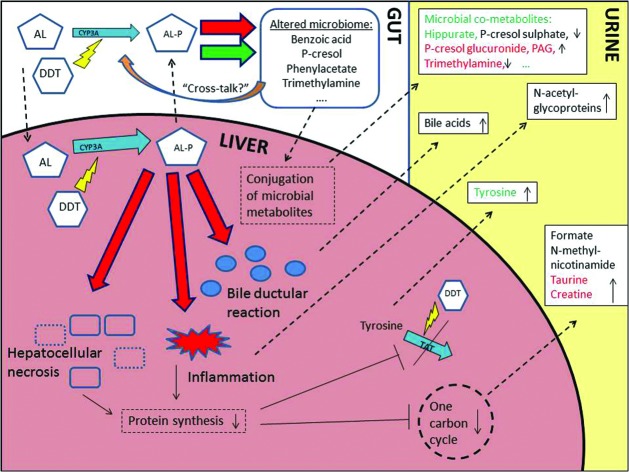
We identified common metabolic effects of pyrrolizidine alkaloid exposure in both humans, following food contamination, and in a mouse model.

## Introduction

Metabonomics/metabolomics, the global profiling of small molecules or metabolites in a biological sample, has been widely applied to the study of liver disease and hepatotoxicity and has revealed important new markers and insight into mechanisms of toxicity and disease.^1^,^2^ It has been proposed as an ideal platform to characterise the “exposome”, the totality of human environmental (*i.e.*, non-genetic) exposures, because it may capture the molecular imprint of exposure through the identification of ‘intermediate markers’ of exposure and disease^3^ and the application of an untargeted approach may identify novel aetiological agents.^4^

During the period 2001–2011, an outbreak of several hundred cases of an often-fatal toxic hepatitis, referred to here as Hirmi Valley Liver Disease (HVLD), occurred in a cluster of rural villages in the North-Western zone of Tigray, Ethiopia.^5^,^6^ It is characterised by epigastric pain and abdominal swelling and appears to be principally caused by exposure to the plant hepatotoxins, pyrrolizidine alkaloids (PAs),^7^ including acetyllycopsamine (AL).^8^ The invasive weed *Ageratum conyzoides* which is highly prevalent in fields of staple crops such as millet, has been identified as the likely source. PAs have caused several outbreaks of severe liver disease worldwide following food contamination and are metabolised in the liver to produce toxic pyrrole metabolites.^9^ The primary liver injury among acute, recent-onset HVLD cases is centrilobular necrosis while among chronic, long-term cases the pathological features include cytomegaly, bile ductular reaction and various stages of fibrosis. Toxicological testing has demonstrated that AL induces similar pathology in mice including centrilobular necrosis and cytomegaly. The residents of the affected villages were also highly exposed to the pesticide DDT (dichlorodiphenyltrichloroethane), due to its use on food grain to protect against storage pests. In the mouse model DDT increased susceptibility to the hepatotoxic effects of AL through induction of the cytochrome P450 enzymes, primarily CYP3A.^8^

Here we have applied ^1^H NMR spectroscopic based metabolic profiling to urine samples collected from HVLD cases and controls. We aimed to identify markers of pyrrolizidine alkaloid induced liver disease that would provide mechanistic insight into the pathogenesis of the disease and that could potentially aid in differential diagnosis. Furthermore, we have explored the utility of this approach as an ‘exposomics’ platform to detect urinary markers of known and unknown toxin exposure. Finally, we aimed to validate and translate our results in humans through a comparative analysis of urine samples collected from mice exposed to both AL alone and a combination of AL and DDT under controlled conditions.

## Experimental

### Sample collection

All experimental protocols were approved and conducted in accordance with the guidelines of Imperial College Research Ethics Committee and were approved by the Minister of Health for Ethiopia and by the Tigray Health Bureau as part of an outbreak investigation. Informed consent was obtained from participants or their parents by local health workers.

Urine samples (*n* = 90) were collected over two visits to the Tigray region. Collections were made during morning visits to clinics at the Kiburto kabelle health post in 2008 and the Kelakil kabelle healthpost in 2009, in a newly-built village for displaced residents of Tseada Amba, the village originally affected by HVLD. 41 subjects that met the pre-defined case definition (abdominal distension, hepatomegaly or splenomegaly on clinical examination and either abdominal pain for at least two weeks or another household member with similar symptoms^8^) and 41 subjects that met the control definition (individuals with no signs of liver disease) were included in the analysis. Furthermore, in the 2009 collection, two cases were hospitalised at the time of collection enabling overnight longitudinal sampling. From one of these patients, four samples were collected and their spectra were included in the analysis, to account for temporal variation. Controls were further classified as either a ‘household control’ (a healthy individual who shares a household with at least one HVLD case), or a ‘village control’ (a healthy individual who lives in a household free from HVLD).

Samples were collected in sterile containers and stored on ice until they were frozen at –20 °C and then transported on ice packs to Imperial College London and stored at –80 °C prior to analysis. The 2009 collected samples were collected into containers pre-filled with the preservative boric acid, while the 2008 collected samples were collected without the addition of preservative. Relative levels of AL in each urine sample were measured by ultra performance liquid chromatography – mass spectrometry (UPLC-MS) as previously described.^8^

### Mouse dosing

All work with mice was approved by UK Home Office and was carried out in accordance with the Animal (Scientific Procedures) Act. Animal husbandry and dosing procedures were as previously described.^8^ Briefly, male C57BL/6J mice, aged 6–10 weeks, were dosed by oral gavage with a racemic mixture of AL (Planta Analytica, USA) dissolved in phosphate buffered saline (PBS). Two experiments were analysed in this study. In an acute AL dosing experiment, eight mice received a single 1500 mg kg^–1^ AL dose and were sacrificed 24 hours later. In an AL and DDT co-dosing experiment, the co-dosed group (*n* = 10) received 75 mg kg^–1^*p*,*p*′-DDT (Sigma-Aldrich, UK, dissolved in olive oil) three times over one week, while the AL-only group (*n* = 10) received the same volume of olive oil only following the same dosing regimen. At the end of the week both groups received 750 mg kg^–1^ AL and were sacrificed 24 hours later. Doses of AL were selected based on pilot dose-range finding studies: the dose used in the acute single dose study was chosen as the dose level required to reproduce the histopathological damage (zonal necrosis) observed in HVLD cases with an acute disease history and symptomology. The dose of *p*,*p*′-DDT used in the co-dosing study was chosen from the literature as the dose level that did not induce any hepatological or neurological effects when administered alone.^10^

Hepatotoxicity was assessed by alanine transaminase (ALT) activity and histopathological examination of plasma and liver samples collected on sacrifice. To grade the magnitude of hepatotoxic effects in the DDT and AL co-dosed group, a histopathological severity score was constructed as follows: 1 = normal, 2 = mild vacuolation/congestion, 3 = hepatocyte swelling, 4 = zone 3 necrosis, 5 = zone 2 and 3 necrosis. Urine samples were collected before dosing in the single dose experiment and on sacrifice in all experiments by free-catch or by extraction directly from the bladder and immediately frozen at –80 °C following the addition of 1% sodium azide as a preservative.

### ^1^H NMR spectral acquisition

Samples were prepared following a standard protocol.^11^ They were first centrifuged at 13 000 rpm and filtered through a 0.2 micron filter. For the human samples, 400 μL of each sample was mixed with 200 μL of phosphate buffer (pH 7.35) prepared with 100% D_2_O solvent (Cortecnet, France), 3 mM sodium azide preservative and 1 mM TSP (3-trimethylsilyl-(2,2,3,3,-^2^H_4_)-1-propionate) (Sigma, UK) as the internal chemical shift reference standard. 550 μL of supernatant was then transferred to a 5 mm NMR tube (Cortecnet, France) for spectral acquisition. For the mouse samples, 30 μL of urine was mixed with 30 μL of the same phosphate buffer and 50 μL of the supernatant was transferred to 1.7 mm NMR capillary samples tubes (Cortecnet, France) housed inside microNMR 5 mm adaptors (New Era, USA) for spectral acquisition.

One-dimensional (1D) ^1^H NMR spectra were acquired using a Bruker Avance II NMR spectrometer (Bruker Biospin, Rheinstetten, Germany) operating at a ^1^H frequency of 600 MHz. A standard 1D solvent suppression pulse sequence was used to acquire the free induction decay (FID; relaxation delay – 90° pulse – 4 μs delay – 90° pulse – mixing time – 90° pulse – acquire FID). The D_2_O present in the buffer provided a field frequency lock, whilst the TSP served as the chemical shift reference compound (*δ*^1^H = 0.00). For acquisition of the human samples 256 scans and 8 dummy scans were collected into 65 000 data points with a spectral width of 12 ppm, relaxation delay of 4 seconds, mixing time of 100 ms and an acquisition time of 4.56 seconds. For acquisition of the mouse samples 128 scans and 8 dummy scans were collected into 65 000 data points with a spectral width of 20 ppm, relaxation delay of 2 seconds, mixing time of 100 ms and an acquisition time of 2.72 seconds. A line-broadening factor of 0.3 Hz was applied prior to Fourier transformation. Spectra were manually phased and baseline corrected using TOPSPIN (version 2.1, Bruker BioSpin).

### Data analysis

Data were imported into the MATLAB computing environment (R2007a, The MathWorks, Inc., MA) and analysed using in-house code. The area between 4.7 and 4.9 ppm was excluded to remove any effect of variation from the suppression of the water resonance. For the spectra from the acute AL mouse dosing study, four separate metabolites of AL, could be identified (ESI†) and these signals were removed from the spectra in subsequent analysis. The spectra were normalised, to account for dilution/concentration differences, using the probabilistic quotient method^12^ and aligned by recursive segment peak alignment.^13^ Data were scaled by mean centring and set to unit variance.

Partial Least Squares Discriminant Analysis (PLS-DA) was applied to model the data and identify discriminatory features based on class membership. For the human samples orthogonal-PLS-DA (O-PLS-DA) was used which incorporates an orthogonal signal correction filter to remove variance not correlated to class membership. All PLS models were first internally validated using 7-fold cross-validation, where portions of the data are subsequently left out and their class membership then predicted to give an indicator of predictive ability, *Q*^2^. Models were then externally validated through random permutation of class membership 1000 times. If the *Q*^2^ of the actual model was in the top 5% of possible *Q*^2^ scores, the model was accepted as valid (*i.e. p* < 0.05 significance level). Similar permutation of the spectral data (10 000 times) was used to assess the significance of metabolites identified as most strongly loading onto the discriminatory component of the PLS-DA models; metabolites with significance level *p* < 0.05 were reported.

Differences in age, urinary AL levels (log-transformed) and duration of disease were assessed by *t*-tests and gender by *χ*^2^ tests. Univariate testing of selected metabolites in the co-treatment mouse model was conducted using Mann–Whitney tests and Spearman's correlations with liver injury.

### Metabolite assignment

Assignment of metabolites responsible for discriminatory peaks was conducted using statistical correlation spectroscopy (STOCSY).^14^ In addition, if necessary, further 2-dimensional NMR experiments were conducted on selected samples on a 600 MHz Bruker Avance II spectrometer. The resultant data was then used to interrogate metabonomic databases such as the Human Metabolome Database^15^ or relevant literature.^16^–^20^

## Results

### Human study

We analysed human urine collections made in 2008 and 2009 separately due to the presence of collection related batch effects. Table 1 summarises the demographics of the study participants: cases in the 2008 collection had on average been ill for less time than the longer term cases surveyed in the 2009 collection (*p* < 0.001). In both collections, a greater proportion of cases were male, although this was significantly different only for the 2009 collection (*p* = 0.004). Participants had a broad age range (4–70 years) with age differences between cases and controls not statistically significant. Cases had higher average levels of urinary AL than controls in both collections (Table 1) although the difference was only significant between cases and village controls in the 2009 collection (*p* = 0.001).

**Table 1:** Demographic information of Hirmi valley liver disease cases and controls, Ethiopia, 2008–2009

Collection	HVLD status	*N*	% Male	Mean age, years (range)	Geometric mean urinary AL, a.u. (95% C.I.)	% Ill less than 1 year	Mean duration of illness, months (range)
Kiburto Health Post 2008	Cases	10	80	23.4 (4–63)	89.9 (45–179.5)	70	13 (2–48)^*a*^
	Controls	7	57	37.6 (18–69)	39.7 (13.5–116.6)	—	—
Kelakil Health Post 2009	Cases	31	60^*b*^	28.8 (4–48)	126.4 (91.9–173.8)^*c*^	6	37 (6–60)
	Household controls	18	11	31.2 (5–70)	84.2 (44.5–159.2)	—	—
	Village controls	17	28	23.7 (4–55)	50.7 (29.1–88.6)	—	—

^*a*^Significantly different compared to 2009 collection cases (*p* < 0.001) based on two-tailed *t*-test.

^*b*^Significantly different compared to village controls (*p* = 0.004) based on *χ*^2^ test.

^*c*^Significantly different compared to village controls (*p* = 0.001) based on one-tailed *t*-test. A.U. = arbitrary units. Duration of illness refers to time since reported onset of symptoms.

**Fig. 1:**
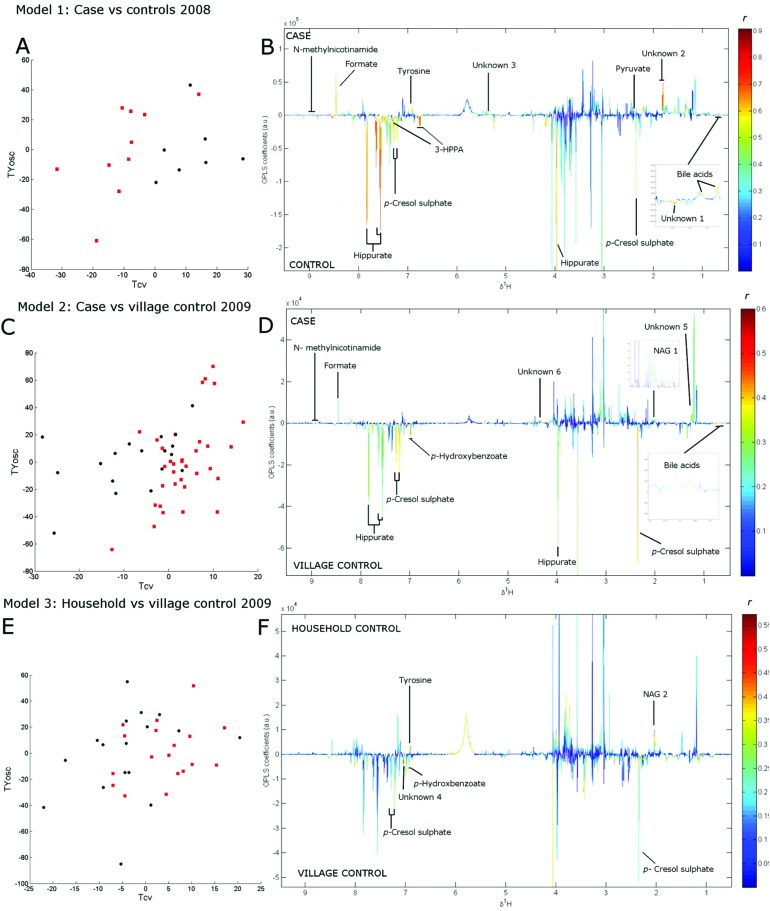
Score scatter plots and back-scaled co-variance loadings plot from human urine samples from Model 1 (A, B). Model 2 (C, D) and Model 3 (E, F). Tcv = cross-validated scores (predictive). TYosc = orthogonal signal corrected scores (orthogonal). Loadings plots are coloured by the absolute loadings correlation coefficient (*r*).

Three separate cross-validated O-PLS-DA models were constructed with the model statistics presented in Table 2 showing the validity of all models. Model 1 compared 10 cases and 7 controls from the 2008 collection with the scores plot given in Fig. 1a, showing clear separation of cases and control by cross-validated predictive scores (Tcv). The loadings coefficient plot for this model showed that concentrations of bile acids, pyruvate, tyrosine, formate and *N*-methylnicotinamide were elevated among cases while concentrations of microbial-associated metabolites *p*-cresol sulphate, hippurate, 3-(3-hydroxyphenyl) propionic acid (3-HPPA) were reduced (Fig. 1b, Table 2).

**Table 2:** Summary of O-PLS-DA models of human samples and discriminatory metabolites

	Model 1	Model 2	Model 3
Collection	2008	2009	2009
Class comparison (N. of samples)	Case (10) *vs*. control (7)	Case (34^*a*^) *vs*. village control (17)	Household control (18) *vs*. village control (17)
Orthogonal components	2	3	2
*Q* ^2^ *Y*	0.48	0.35	0.20
*R* ^2^ *Y*	0.98	0.97	0.96
*R* ^2^ *X*	0.33	0.29	0.22
Permutation *p* value	0.035	0.001	0.041

Metabolite	Loading correlation coefficient (*r*)		
Bile acids	0.67**	0.50**	0.12
*p*-Cresol sulphate	–0.57**	–0.39**	–**0.31**
Pyruvate	0.62**	—	–0.06
Hippurate	–0.75**	–0.33**	–0.24
3-HPPA	–0.69**	–0.07	0.04
Tyrosine	0.62**	0.22	0.46**
Formate	0.55*	0.29	0.16
*N*-Acetylglycoprotein 1	0.14	0.41**	0.14
*N*-Acetylglycoprotein 2	—	—	0.45*
*p*-Hydroxybenzoate	—	–0.42**	–0.45
*N*-Methylnicotinamide	**0.42**	0.30**	0.03
Unknown 1	–0.75**	—	—
Unknown 2	0.76**	—	—
Unknown 3	0.75**	—	—
Unknown 5	0.01	0.40**	0.19
Unknown 6	0.14	0.44**	0.15

^*a*^Four longitudinal samples from one case included. 3-HPPA = 3-(3-hydroxyphenyl) propionic acid.

Model 2 compared spectra from 31 cases (including four longitudinal spectra from one individual) and 17 village controls from the 2009 collection (Fig. 1c). Since household controls shared the same food supply as cases, and were therefore likely to have higher long-term PA exposure than village controls, we excluded household controls from the analysis in model 2 to improve contrast between classes. The loadings coefficient plot of model 2 (Fig. 1d) showed that the relative levels of bile acids, *N*-acetylglycoproteins and the methyl acceptor *N*-methylnicotinamide were found to be higher in samples from cases than in samples from village controls, while the concentration of *p*-cresol sulphate, hippurate and another microbial associated metabolite *p*-hydroxybenzoate were relatively lower in case samples (Table 2).

The effects of differential exposure levels among controls was explored in model 3, which compared 17 village to 18 household control samples collected in 2009 (Fig. 1e). The loading coefficient plot of model 3 (Fig. 1f) showed that the relative concentration of tyrosine and *N*-acetylglycoprotein (although a different glycoprotein resonance than that identified in model 2) was greater and the relative concentration of *p*-cresol sulphate and *p*-hydroxybenzoate was lower among household control samples (Table 2).

**Fig. 2:**
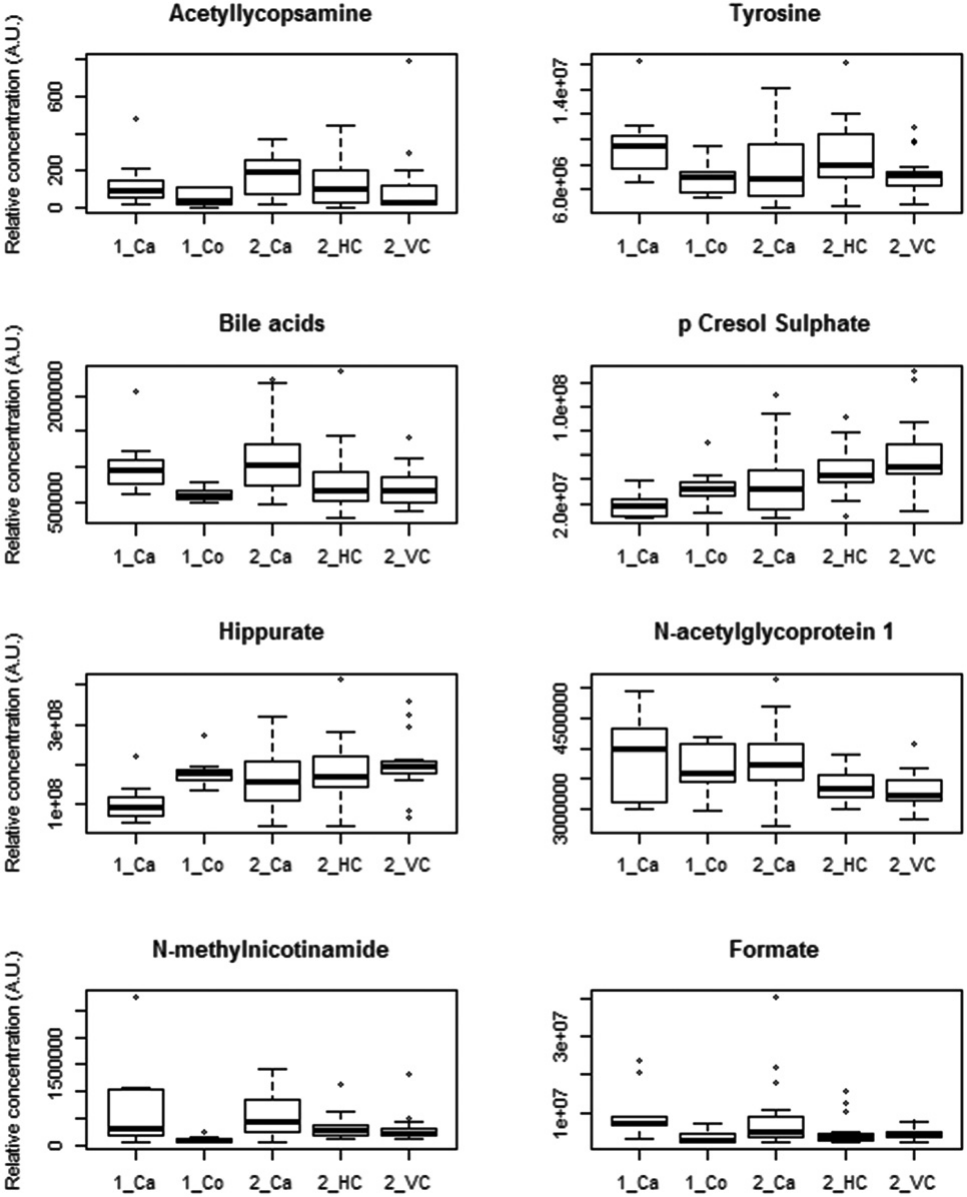
Box and whisker plots showing the relative distributions of acetyllycopsamine and endogenous discriminatory metabolites across sample classes. Thick black line = median, box = interquartile range, circles = outlier values. Class category key: 1_Ca = case from 2008 collection; 1_Co = control from 2008 collection; 2_Ca = case from 2009 collection; 2_HC = household control from 2009 collection, 2_VC = village control from 2009 collection. Note that relative concentration of acetyllycopsamine is not directly comparable to concentrations of other metabolites.

Similar trends in the relative concentration of the discriminatory metabolites bile acids, *N*-acetylglycoproteins (identified in model 2), hippurate, *p*-cresol sulphate, formate and *N*-methylnicotinamide were observed across the sample classes in both collections (Fig. 2). NMR spectra and loadings coefficient plots showed the presence of singlets from bile acid C-18 methyl groups in models 1 and 2, which were likely to arise from taurine-conjugated or unconjugated species.^16^ A number of metabolites that remain unidentified were also perturbed in each model (Table 2).

Due to the uneven gender distributions between cases and controls, a further O-PLS-DA model was constructed to assess the metabolome relationship with gender. Of the metabolites identified above, only *p*-cresol sulphate levels were found to be associated with gender with lower levels in samples from male subjects (loading coefficient *r* = –0.15) However the association of this metabolite with case samples was robust to adjustment for gender in multiple logistic regression models (see ESI†).

### Murine model of acute AL toxicity and AL and DDT co-treatment

Full histopathological and clinical chemistry results from the two mouse experiments have been presented previously.^8^

In the single AL dose study, 6/8 mice had plasma ALT levels above 1000 U L^–1^ (upper limit of assay) 24 hours post-dose. Hepatocellular necrosis of the zone 3 region, sometimes extending into zone 2, of the liver lobule was observed in 7/8 mice. A PLS-DA model (*R*^2^*Y* = 0.75, *R*^2^*X* = 0.33, *Q*^2^*Y* = 0.61, validation *p* = 0.001) was applied to distinguish urinary metabolic profiles from six pre-dose samples and five post-dose samples available in this study. The metabolites significantly contributing to the discriminatory component included amino acids creatine (loading *r* = 0.93), taurine (loading *r* = 0.84) and tyrosine (loading *r* = 0.72) which were raised 24 h post dose. The microbial associated metabolites hippurate (loading *r* = –0.80) and trimethylamine (loading *r* = –0.69) were decreased, while phenyacetylglycine (loading *r* = 0.79) and *p*-cresol glucuronide (loading *r* = 0.79) were elevated post dose.

In the AL and DDT co-dosing study, mean plasma ALT levels were 85 U L^–1^ (Standard Error (SE): 16) in the AL only dosed group and 470 U L^–1^ (SE: 205) in the AT + DDT co-dosed group. In the AL only dosed group histopathological damage was limited to swollen hepatocytes in the zone 1 region in one mouse. In the AL + DDT co-dosed group histopathological features included no observable injury (1/10 mice), mild vacuolation (2/10 mice), zone 3 congestion (1/10 mice), swollen hepatocytes in the zone 3 region (3/10 mice), extensive zone 3 necrosis (1/10 mice), extensive zone 2 and 3 necrosis (2/10 mice).

**Fig. 3:**
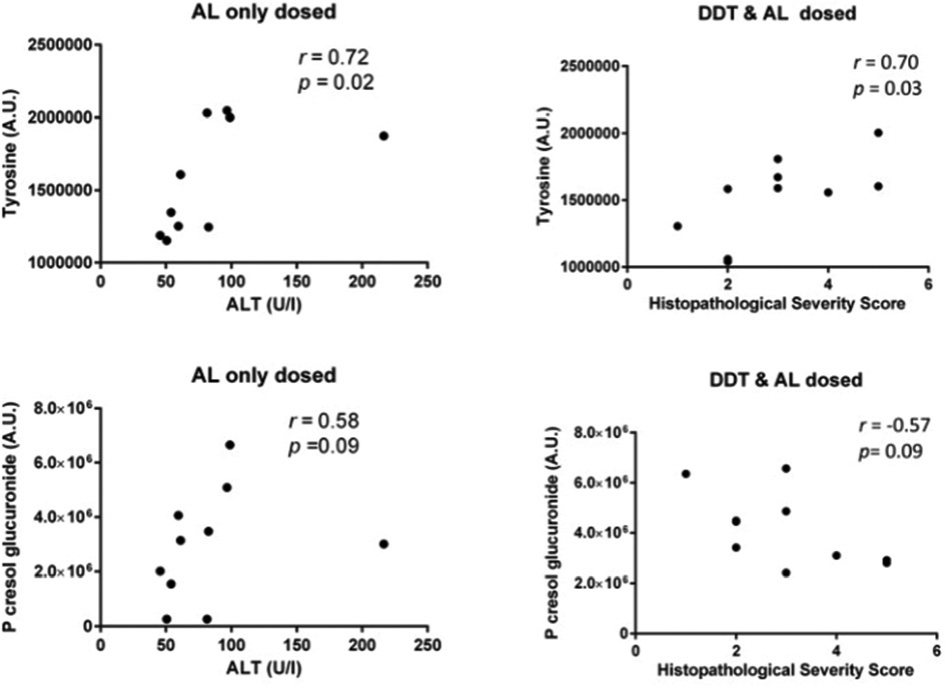
Spearman's correlations between tyrosine and *p*-cresol glucuronide and extent of liver injury in AL and DDT co-dosing study. Left hand side: Correlations within AL only dosed group. Right hand side: Correlations within DDT + AL co-dosed group. Top: Correlations with tyrosine. Bottom: Correlations with *p*-cresol glucuronide. ALT = alanine.

Since multivariate methods could not clearly distinguish samples from the two dosing groups in the DDT and AL co-treatment study (data not shown), the relative urinary concentrations of endogenous metabolites identified as being perturbed following acute AL dosing were compared between mice in the AL-only and DDT + AL dosed groups (ESI Fig. s4†). No significant differences were observed between the groups, although the differences in median concentrations were greater for metabolites associated with gut microfloral co-metabolism than for metabolites associated with liver injury. The largest difference was observed for hippurate, which had a lower median concentration among DDT + AL treated mice, but this was not statistically significant (*p* = 0.075). Despite similar median levels in both dosing groups, tyrosine was correlated with the extent of liver injury (Fig. 3) in both the AL only dosed group (*r* = 0.72, *p* = 0.02, with alanine transaminase (ALT) score) and the DDT + AL dosed group (*r* = 0.70, *p* = 0.03, with histopathology score). Interestingly, *p*-cresol glucuronide was positively correlated with liver injury in the AL only dosed group (*r* = 0.58, *p* = 0.09, with ALT score) but negatively correlated in the DDT + AL dosed group (*r* = –0.57, *p* = 0.09, with histopathology score) although this was not significant at the 5% level.

## Discussion

We have presented a unique application of untargeted metabolic phenotyping to a novel human liver injury caused by the plant hepatotoxins, pyrrolizidine alkaloids. HVLD cases and controls were distinguished through multivariate analysis of their urinary NMR spectroscopic metabolic profiles, across two different sample collections. Furthermore, apparently healthy controls were distinguished based on their household, a proxy for long-term toxin exposure. A number of metabolites were associated with disease and/or exposure status, broadly grouped into those reflecting liver function (tyrosine, bile acids), one-carbon metabolism (*N*-methylnicotinamide, formate) and the gut microbiome (hippurate, *p*-cresol sulphate). Despite differences between sample collections in terms of location, overall urinary AL levels and disease duration, similar metabolic changes were observed across the different comparisons. Disease duration (*i.e.* the time since reported onset of illness) reflects the different stages of HVLD, with patients in the early stages of the disease most likely to suffer from the acute form of the disease, characterised by sudden hepatomegaly and ascites, abdominal pain and bleeding abnormalities, raised serum amino transferase levels and hepatocyte necrosis. Patients ill for longer than a year are most likely to have moved into the chronic stage of the disease, which in many cases is indistinguishable from other forms of chronic liver disease such as compensated cirrhosis.^8^ We observed some differences in metabolic response between the two collections that may be attributed to differences in disease staging. For instance, elevations in tyrosine levels were not statistically significant among the more chronic cases of the second collection. Tyrosine levels were significantly raised however, among the apparently healthy household controls compared to village controls that had lower exposure levels. The household controls appeared unaffected by HVLD, and this could be attributed to within household differences in dietary practices providing some reduction in long-term PA exposure and/or inter-individual differences in toxin susceptibility.

We sought to validate our results through similar analysis of urine samples collected from mice following an acute single dose of AL. Again metabolites falling into similar metabolic pathways as observed in the human comparisons; liver-associated (tyrosine), one-carbon metabolism (taurine, creatine,) or gut microbial (hippurate, *p*-cresol glucuronide, phenylacetylglycine and trimethylamine), were perturbed in the mice models. Furthermore, levels of specific metabolites, tyrosine and hippurate, were perturbed in both the human and mouse analyses reflecting similar metabolic responses to PA exposure. Since the population affected by HVLD were also highly exposed to DDT, we also tested the effect of prior dosing with DDT on the metabolic response to AL dosing. Despite greater hepatotoxicity in the DDT pre-dosed group, there were only small differences in metabolic response, suggesting a ‘saturation effect’ for markers such as taurine and creatine, with only little change past a certain level of liver damage. However, for tyrosine and *p*-cresol glucuronide, which were more closely related to the extent of hepatocellular damage, there was evidence for modulation of the metabolic response following DDT pre-dosing.

**Fig. 4:**
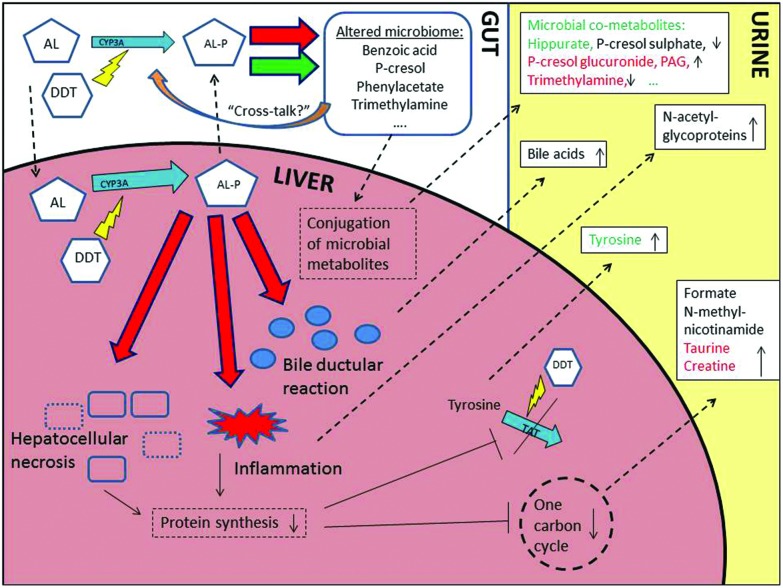
Summary of postulated effects of exposure to acetyllycopsamine (AL) in humans and mice, as detected by changes to urinary 1H NMR spectroscopic metabolic profile. Blue arrows show enzymatic reactions. Red arrows show cytotoxic effects. Green arrow shows potential beneficial effects on certain microbial species (*via* reduced competition with AL sensitive species or alternate metabolic routes). Orange arrow show potential “cross-talk” with AL metabolism (*e.g. via* induction of or competition for host toxification/detoxifying enzyme systems). Dotted arrows show movement of metabolites between compartments (the blood compartment is not shown for simplicity). Square white boxes show metabolites with perturbed urinary levels. Vertical arrows show increase or decrease in liver processes or urinary levels of metabolites. Colour of text of urinary metabolites indicates whether detected in human models (black), mouse models (red) or both mouse and human (green). Yellow lightning bolts indicate inductive effects. DDT = dichlorodiphenyltrichloroethane, AL-P = bioactive pyrrole metabolite (s) of AL, TAT = tyrosine aminotransferase.

Fig. 4 summarises the novel panel of discriminatory metabolites identified across the human and mouse studies and relates them to the known effects of AL exposure. Tyrosine elevations were observed in both mouse and human models with the more acute hepatocellular injury. Hypertyrosemia is often observed following chronic liver diseases^21^ and it is normally accompanied by rises in other aromatic amino acids such as phenylalanine reflecting reduced hepatic metabolism. However, in this study no changes were observed in phenyalanine indicating the changes in tyrosine appear to be a toxin specific response rather than a result of general liver insufficiency. It is likely that AL interrupts the ability to re-synthesise the short lived enzyme tyrosine aminotransferase (TAT) involved in tyrosine catabolism.^22^ The dampening effect on the tyrosine response, considering the increased hepatocellular injury, when DDT was co-administered would support this interpretation since phenobarbital-type inducers are known to up-regulate TAT production.^23^

One-carbon metabolism intermediaries have been suggested as useful probes of liver specific processes.^24^ Rises in the methyl-acceptor *N*-methylnicotinamide, the one carbon hub metabolite formate, and also taurine, an end-product of sulphur metabolism, may reflect a fall in one-carbon cycling due to reduced cysteine demand following an overall drop in protein synthesis. When elevation of taurine is accompanied by a rise in creatine, as observed following acute AL dosing, this is thought to relate to active synthesis due to the cytoprotective properties of taurine.^25^

Increases in resonances from the *N*-acetyl group of the acute phase proteins α_1_-acid glycoproteins, were detected among both disease cases and highly exposed controls. These NMR spectral resonances are becoming established as indicators of systemic inflammation in many disease contexts.^26^ The rises observed in bile acids appear to result from specific molecular forms rather than resulting from general cholestasis, which was a minor component of HVLD. Specific changes to bile acid profiles have been detected by targeted UPLC-MS and have been associated with specific forms of liver injury,^27^ and may reflect the observed bile ductular reaction in cases or host – gut microfloral interaction.

Lower levels of gut-microbial associated metabolites, including hippurate and *p*-cresol sulphate, were observed in case samples. Reductions in *p*-cresol sulphate have not been reported in studies of liver disease in humans previously, indicating the importance of unique microbial profiles in HVLD. Hippurate is produced by the conversion of dietary aromatic compounds by bacteria such as Clostridia species to benzoic acid, followed by glycine conjugation in the kidney or liver.^28^ The mouse model supports that, for hippurate, lower levels among HVLD cases are a result of toxin exposure. However, rises observed in AL-only dosed mice of phenylacetylglycine and *p*-cresol glucuronide, the rodent conjugated form of *p*-cresol, suggests that some microbial species in mice are resistant to AL exposure or that conjugation capacity is altered by AL exposure. Modulation of host xenobiotic metabolic machinery by the microbiome is now well established either through, for example, direct enzyme induction^29^ or competition for conjugation capacity.^30^ It is conceivable therefore, that the higher levels of *p*-cresol sulphate observed in controls compared to cases in the human studies may, in part, precede and modulate the effects of AL exposure. The trend for reduced levels of *p*-cresol glucuronide with increased hepatotoxicity in mice co-dosed with both DDT and AL suggests additional complexity in the toxin-host-microbiome relationship.

Limitations of this study were a lack of accompanying traditional clinical chemistry measures in the patient samples to assess the severity of disease within classes and strengthen the disease classification. While the majority of cases in the first collection were confirmed by liver function test (*i.e.* raised serum γ-glutamyl transpeptidase activity^8^) or biopsy, the confirmation by liver function test in the second collection was not logistically possible. This may have led to misclassification of some cases (and controls) in the analysis of samples from the second collection, although the same specific case definition was used in both collections. Urinary AL measurements only reflect recent exposure, and the use of other markers such as pyrrole-protein adducts would have proved an assessment of long-term, although non-specific, PA exposure. It was also not feasible to completely age and gender match cases and controls, although potential confounding has been adjusted for. Specific information on diet was not obtained, and diet is known to affect urinary metabolic profiles. However, the relative homogeneity of the diet of the participants, relative to western populations, negates the importance of this to some extent. Furthermore, our sample size was relatively small. A larger sample size may have allowed the use of other statistical approaches such as multiple univariate testing with correction for multiple testing. Here we have used a single multivariate modelling approach, partially to increase statistical power and avoid the multiple testing burden. While the study was sufficiently powered to detect differential metabolic phenotypes, future studies may consider increasing sample size to detect additional associations. The NMR platform used has limited sensitivity, and may therefore have been unable to detect metabolites of very low concentration that had aetiological or diagnostic relevance. However, the reproducibility of the platform and the structural information it provides makes it particularly suitable for ‘untargeted’ analyses. Finally, the cross-sectional nature of the human study provided only a ‘snapshot’ without information regarding the temporal sequence of metabolic changes and clinical disease onset. However, this was addressed to some extent by the analysis of differentially exposed healthy controls and the integration of mouse models, which provided both validation and further information on the causal direction of detected associations. Future work may include adopting a ‘One Health approach’ and performing similar analyses in livestock of the affected villages, that were similarly affected by a parallel outbreak of liver disease.

## Conclusions

This is the first metabolic phenotyping study of hepatotoxin-induced disease in humans. Untargeted global ^1^H NMR analysis has been used to distinguish both HVLD cases from controls; and healthy controls differentially exposed to AL. A number of metabolites associated with liver dysfunction have been identified alongside indicators of alterations to the gut microbiome. Tyrosine and *p*-cresol sulphate were found to be sensitive indicators of exposure and early stage disease, while changes to the concentrations of hippurate, formate, bile acids, *N*-acetylglycoproteins and *N*-methylnicotinamide, were found to be indicative of both acute and chronic stages of HVLD. Furthermore, common metabolic pathway changes and perturbation of specific metabolites were identified in an integrated pre-clinical murine model. Pathways identified here may be useful as “exposome” signals and in translational research, for example, these markers may have potential to be developed into simple clinical tests to aid differential diagnosis of PA induced liver disease in resource-poor countries.

## Conflict of interest

There are no conflicts of interest to declare.

## Supplementary Material

Supplementary information
