# Supplementary material for: Global metabolic changes induced by plant-derived pyrrolizidine alkaloids following a human poisoning outbreak and in a mouse model
Source: Toxicol Res (Camb). 2016 Aug 12;5(6):1594–603. doi: 10.1039/c6tx00221h (PMC6060677; doi:10.1039/c6tx00221h)
Supplement: Supplementary file 1 [file TX-005-C6TX00221H-s001.pdf]

Electronic Supplementary Material (ESI) for Toxicology Research.
This journal is © The Royal Society of Chemistry 2016

Supplementary Information

Global metabolic changes induced by plant-derived pyrrolizidine alkaloids following a human poisoning outbreak and in a mouse model.

Oliver Robinson, Mireille B. Toledano, Caroline Sands, Olaf Beckonert, Elizabeth Want, Rob Goldin, Mike Hauser, Alan Fenwick, Mark Thursz, Muireann Coen.

Contents

Effects of Gender on metabolic profile 1
Identification of AL metabolites 24 hours after single 1500 mg/kg dose AL 3
Univariate analysis of selected endogenous metabolites in AL and DDT co-dosing study 4

Effects of Gender on metabolic profile

To determine whether there was any confounding effect by gender on the O-PLS-DA models presented comparing disease class, an O-PLS-DA model comparing gender was constructed (model statistics: $R^2Y=0.85$, $R^2X=0.19$, $Q^2Y=0.24$). The most important contributor to the predictive component was *p*-cresol sulphate (loading correlation coefficient r = -0.15 with predictive component for being male), which had been identified as contributing to the separation of metabolic profiles in the main analyses (table 2). No other metabolites were indentified in the O-PLS-DA model for gender that had been identified as discriminatory in the main analysis models.

The distribution of *p*-cresol sulphate concentrations across sample classes, when stratified by gender, was broadly similar to previously found (Fig. S1), with median concentrations in case samples lower than the median concentrations of their respective control samples. To adjust statistically for any gender bias in the association between *p*-cresol sulphate and HVLD, a linear regression model was constructed to assess the relationship between levels of *p*-cresol sulphate (log transformed) and case or control status while adjusting for gender (table s1). When all available case samples (N= 44)

were regressed against all available control samples (N=42), a significant effect was still observed after adjusting for gender.

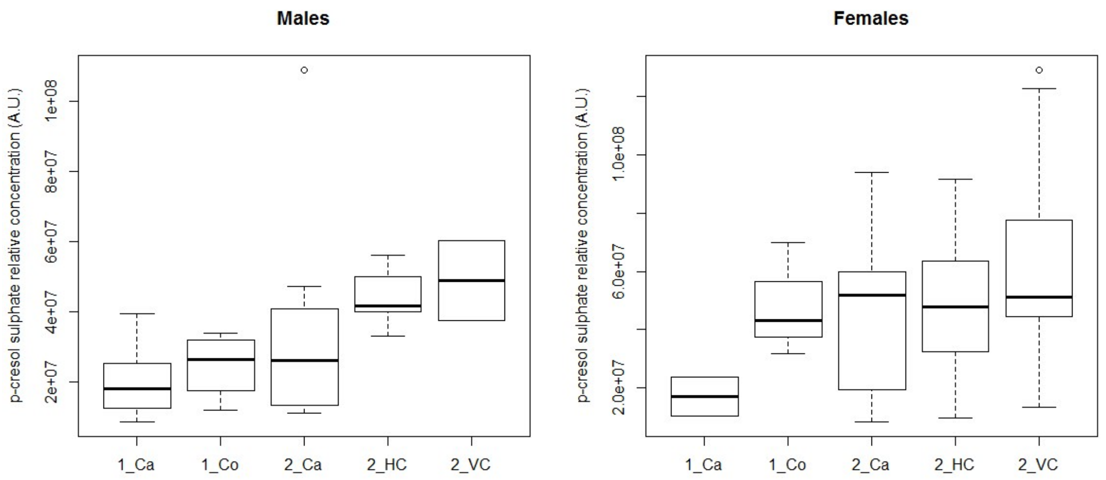

Figure s1: Box and whisker plots showing the relative distributions of *p*-cresol sulphate across sample classes, for male and female samples. Thick black line = median, box= interquartile range, circles =outlier values. Class category key: 1_Ca= Case from 2008 collection; 1_Co= Control from 2008 collection; 2_Ca = Case from 2009 collection; 2_HC= Household control from 2009 collection, 2_VC = Village control from 2009 collection.

Table s1: Summary of linear regression model of relationship between p cresol sulphate relative concentration (a.u., log transformed) and HVLD case/control status and gender.

	β coefficient (95% confidence interval)	P value
HVLD case	-0.41 (-0.68 to -0.13)	0.004
Male	-0.33 (-0.61 to -0.05)	0.022
Intercept	176.92 (17.49 -17.89)	0.000

Identification of AL metabolites 24 hours after single 1500 mg/kg dose AL

Metabolites of AL were detectable in the urine of mice 24 hours after a 1500 mg/kg dose (figure s2). Although the metabolites were not conclusively identified, STOCSY analysis [1] and 2D NMR was used to determine the interconnectivity of the AL metabolite signals and was able to separate them into four different groups, each arising from a different molecule (table s2). Metabolite AL-3 was tentatively identified as an AL-glucuronide conjugate, as confirmed by the presence of the doublet signal at 5.24 ppm (J-coupling 3.76 Hz) arising from the anomeric proton of the glucuronide moiety (figure s2).

Table s2: Chemical shifts of the four acetyl-lycopsamine metabolites detected in mouse urine 24 hours after a 1500 mg/kg dose, determined by STOCSY analysis. Multiplicity of signal is annotated if identifiable (s=singlet, d=doublet, m=multiplet).

Metabolite	Chemical shifts (ppm)
**AL-1**	0.88m, 1.615m, 1.875, 2.298, 3.68, 4.125, 5.074, 7.058
**AL-2**	0.99, 1.15, 1.1d, 1.38d, 3.96
**AL-3**	1.003, 1.33d, 1.494, 2.14, 3.23, 3.75, 4.134, 5.24d
**AL-4**	0.89, 2.238, 4.26, 4.439, 4.912, 5.805, 5.983, 8.558

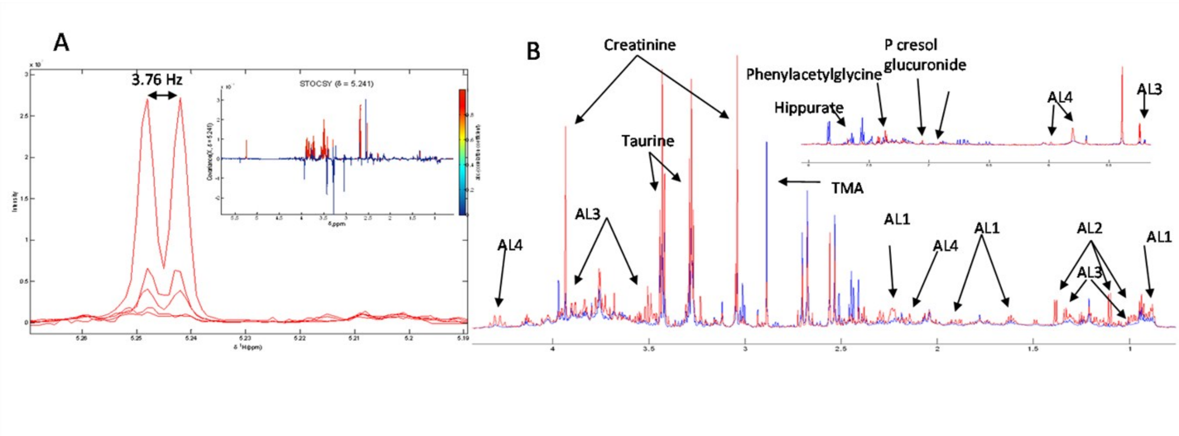

Figure s2: ^{1}H NMR urinary spectra of mice 24 h post 1500 mg/kg dose of AL. A: Post dose spectra showing doublet signal at 5.24 ppm from glucuronide moiety of metabolite AL-3, annotated with J-coupling. Inset shows STOCSY diagram with peaks structurally correlated (*r*=1) to this region showed in red. B:Mean of pre-dose spectra (blue) overlaid with mean the mean of post-dose spectra (red). Discriminatory endogenous metabolites AL metabolites are annotated

Univariate analysis of selected endogenous metabolites in AL and DDT co-dosing study

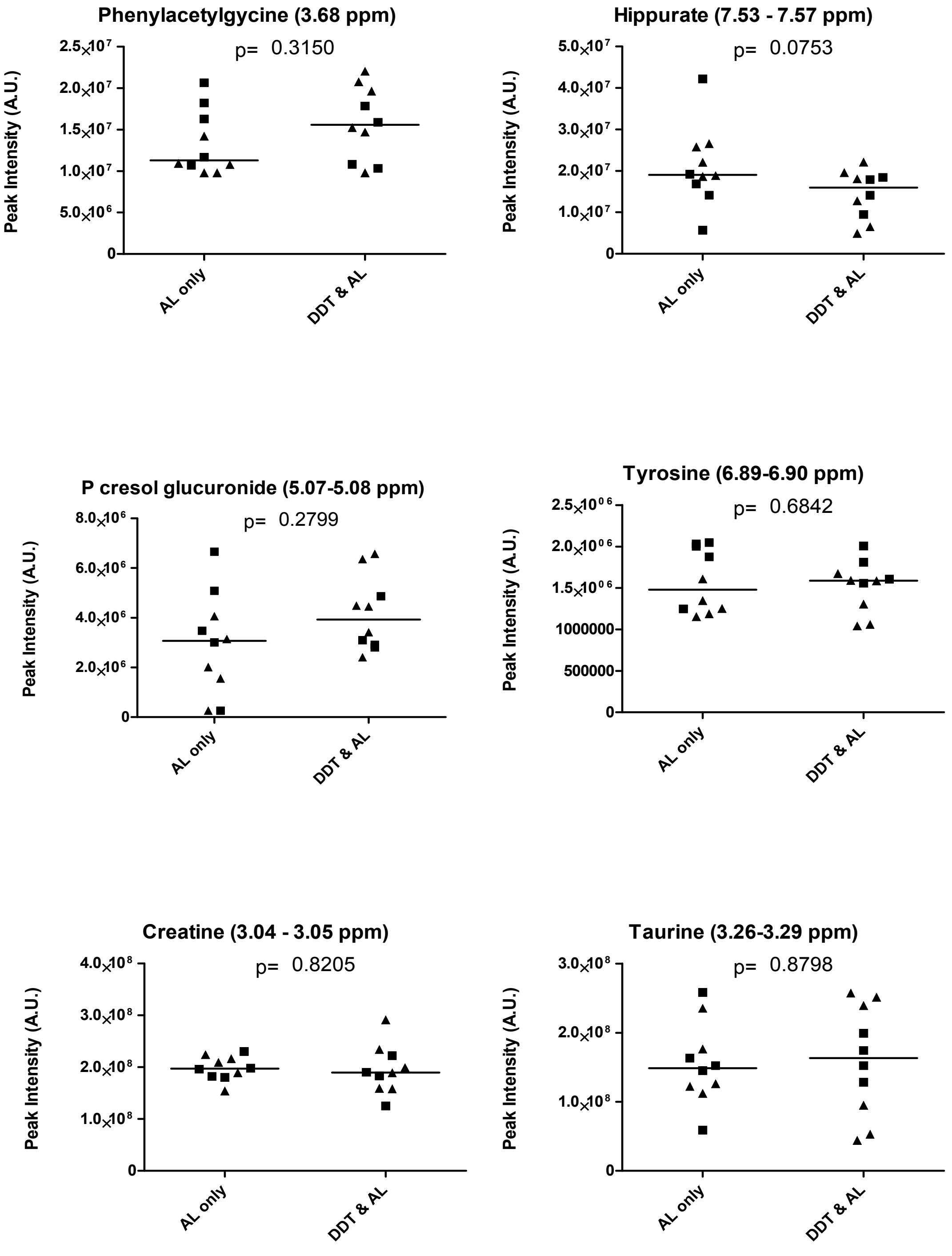

Figure s3: Comparison of urinary concentration of selected endogenous metabolites between the AL only and the DDT and AL treated groups. Titles indicate the region of the spectrum integrated to derive metabolite peak intensities. Points represent values for individual mouse samples. Horizontal bars show median values. P values calculated from Wilcox Rank Sum test comparing groups.

Reference

1. Cloarec, O., et al., *Statistical total correlation spectroscopy: an exploratory approach for latent biomarker identification from metabolic 1H NMR data sets.* Anal Chem, 2005. **77**(5): p. 1282-9.
